# Alcohol and Female Puberty: The Role of Intraovarian Systems

**Published:** 2001

**Authors:** W. Les Dees, Vinod K. Srivastava, Jill K. Hiney

**Affiliations:** W. Les Dees, Ph.D., is a professor, Vinod K. Srivastava, Ph.D., is a research assistant professor, and Jill K. Hiney, Ph.D., is a research associate in the Department of Veterinary Anatomy and Public Health at Texas A&M University, College Station, TX

**Keywords:** puberty, female, reproductive effects of AODU (alcohol and other drug use), ovarian function, estradiol, secretion, insulin-like growth factor, binding proteins, nitric oxide, underage drinking

## Abstract

Alcohol consumption during early adolescence may suppress the secretion of specific female reproductive hormones, thereby delaying puberty and adversely affecting the maturation of the reproductive system. These effects occur through several mechanisms, including altered production and secretion by the ovaries of estradiol, a key steroid hormone involved in the timing and regulation of female reproductive events. Alcohol can affect estradiol production by interfering with the normal function of regulatory hormones produced by the brain and the pituitary gland. Recent research has demonstrated additional potential mechanisms for alcohol’s effects on female reproductive capability, including interference with specific regulatory systems located entirely within the ovary. Such “intraovarian” systems include the insulin-like growth factor-1 (IGF-1) and nitric oxide (NO) systems. Alcohol can dampen the stimulatory effects of the ovarian IGF-1 system and can increase the inhibitory effects of the ovarian NO system. These effects combine to decrease estradiol secretion. Thus, alcohol impairs ovarian function not only by interfering with hormonal communication between the brain, pituitary gland, and ovaries but also by directly altering the function of regulatory systems within the ovaries themselves. These results provide further evidence of the risks of underage drinking and the importance of its prevention.

The rapid physiological changes that occur during early adolescence are vulnerable to alcohol’s effects, potentially leading to long-term consequences. Alcohol’s ability to interfere with normal reproductive function in women (Hiller-Sturmhöfel and Bartke 1998) suggests that alcohol use during late childhood may adversely affect female puberty. This possibility is supported by studies showing that mildly intoxicating doses of alcohol can inhibit the secretion of puberty-related hormones in young female rats and rhesus monkeys, thereby delaying sexual maturation (Dees and Skelley 1990; Dees et al. 2000). Limited research also has revealed low levels of reproductive hormones in alcohol-abusing adolescents (Diamond et al. 1986; Block et al. 1993). To study alcohol’s effect on female puberty, scientists must understand the complex events that initiate normal sexual maturation.

Female sexual development is regulated by timely changes and complex interactions among specific hormones and chemical messengers. These substances originate primarily from three sites: (1) the hypothalamus, a part of the brain that controls pituitary hormonal secretions; (2) the pituitary gland, located immediately below the hypothalamus, which produces specific hormones that influence the reproductive cycle and other major physiological functions; and (3) the ovaries, paired organs in the pelvic area that produce the egg cells and female steroid hormones. The ovarian steroid hormone of primary importance to sexual function is called estradiol. The three regulatory sites described above are known collectively as the H-P-O axis.

In addition to the effects of pituitary hormones on ovarian development and steroid production, evidence reviewed here supports an important regulatory role for specific intraovarian control mechanisms. The term “intraovarian” refers to the presence, entirely within the ovary, of (1) the cell types required to produce a given substance, and (2) the appropriate “target” cells or cell components that respond to the substance by performing or triggering the desired biological action.

The hormone insulin-like growth factor-1 (IGF-1) and the chemical messenger nitric oxide (NO) fulfill these criteria and operate within the ovary through two complete and independent systems.^1^ Both IGF-1 and NO are known to influence a wide variety of functions throughout the body. IGF-1 supports the effects of growth hormone (GH) by stimulating cell proliferation and can induce the release of specific puberty-related hormones (for reviews, see Daughaday and Rotwein 1989; Hiney et al. 1996). Because NO exists in gaseous form and dissipates quickly, it must exert its actions at or near the site where it is produced. NO’s many physiological roles are still under study. Because the ovary is the major site of prepubertal estradiol production, and because both the IGF-1 and NO systems can influence ovarian steroid hormone production (Adashi 1993; Olson et al. 1996), it is important to understand the effects of alcohol on their respective ovarian actions. This article first provides background information on the regulation of female puberty and then describes the components of the intraovarian IGF-1 and NO systems, explores alcohol’s effects on these systems and their significance in terms of normal reproductive capacity, and suggests proposed mechanisms by which alcohol may alter the functioning of these two intraovarian systems.

## A Brief Overview of Puberty

The midpoint of a woman’s monthly reproductive cycle is marked by ovulation, the release from the ovary of a single fully developed egg cell. Reproduction becomes possible at first ovulation (i.e., puberty), which generally occurs between the ages of 10 and 14. As female puberty approaches, the pituitary gland begins to secrete increasing amounts of luteinizing hormone (LH), follicle stimulating hormone (FSH), and GH into the bloodstream in response to stimulatory “releasing” hormones produced in the hypothalamus (see figure). The increased levels of these three hormones promote maturation of the ovaries. As the ovary matures, it produces and secretes increased amounts of the principal female hormone, estradiol. Released into the blood, estradiol exerts multiple actions in the body; for example, estradiol determines the overall pattern of body fat distribution, which results in the typical female body shape. Upon reaching the brain, estradiol helps stimulate the final maturation of the hypothalamus so that it can drive the pubertal process until first ovulation. Following puberty, LH and FSH continue to help regulate the events of the monthly female reproductive cycle. For a more detailed review of puberty-related events, see Dees and colleagues (1998).

## Overview of the Ovarian IGF-1 System: Components and Actions

Evidence suggests that some of the actions of GH on the ovary may be mediated by increased intraovarian production of IGF-1 (Yoshimura et al. 1994). An intraovarian IGF-1 system has been found in every animal species in which it has been sought. This system consists of three main components, all of which interact to elicit or modify the biological actions of IGF-1 within the ovary:

- The first component is IGF-1 itself, a 70-amino acid peptide (basically a small protein). Although the sites and levels of production of IGF-1 vary, IGF-1 itself has been found in ovarian tissues of the rat (Adashi et al. 1985), sheep (Monniaux and Pisselet 1992), pig (Baranao and Hammond 1984), cow (Schams et al. 1988), and human (Erickson et al. 1989). Only the liver and uterus produce more IGF-1 than does the ovary.
- The second component is the type-1 IGF receptor (IGF-1R). Receptors are specialized proteins, generally embedded in a cell’s outer membrane, that recognize specific chemical messengers. The IGF-1 receptor strongly binds the IGF-1 peptide in the cell membrane, thereby triggering physiological processes that ultimately elicit IGF-1’s physiological action (see following text).
- The third component consists of a family of six similar but distinguishable IGF-binding proteins (IGFBPs), which, although completely distinct from IGF-1R, can also bind IGF-1 and alter its function.

Besides promoting the growth and multiplication of cells, IGF-1 may contribute to ovarian development by promoting the actions of LH and FSH (Adashi 1993; Ying and Zhang 1999). However, IGFBPs can influence the concentration of IGF-1 and its interactions with the IGF-1 receptor, potentially inhibiting the function of ovarian cells (Adashi 1993, Hammond 1999).

## Effects of Alcohol on the Ovarian IGF-1 System

Researchers have hypothesized that alcohol may alter prepubertal ovarian physiology at least partly through its effects on the intraovarian IGF system. Srivastava and colleagues (1999) found that chronic (i.e., 5-day) alcohol exposure activates the first steps of IGF-1 production (i.e., gene expression) in rats. Gene expression begins when the genetic blueprint for producing a specific peptide is “decoded” and the information is copied onto molecules called mRNA. The mRNA passes this information on to specialized parts of the cell where finished peptides as well as full-length proteins are assembled. Interestingly, the elevated levels of IGF-1 mRNA were accompanied by *decreased* levels of IGF-1 itself within the immature ovary. An observed decline in blood estradiol levels was consistent with decreased IGF-1 availability (Srivastava et al. 1999).

Although IGF-1 production in most tissues is typically stimulated by GH, alcohol has been found to decrease GH levels in humans (Ganda et al. 1978) as well as in animals (Redmond 1980; Dees and Skelley 1990; Emanuele et al. 1992). However, alcohol does not suppress secretion of FSH (Dees and Kozlowski 1984; Dees et al. 1985; Dees and Skelley 1990), which is 3 times more potent than GH at initiating IGF-1 gene expression (Samaras et al. 1996). Thus, increased IGF-1 mRNA levels in alcohol-treated rats may result from continued stimulation by FSH. Alcohol itself may subsequently suppress formation of the finished IGF-1 peptide through some unknown process. Together, these events would lead to the accumulation of IGF-1 mRNA with a reduction in finished IGF-1 peptide levels. Alcohol’s effect on IGF-1 formation cannot be attributed to a generalized suppression of protein synthesis, because not all protein levels in the ovary decrease in response to alcohol (Srivastava et al. 1999).

Srivastava and colleagues (1999) also studied alcohol’s effects on the ovarian IGF-1 receptor protein. In rats, 5 days of exposure to alcohol decreased ovarian IGF-1 receptor gene expression with decreases in *both* the mRNA and the finished protein. Although the mechanism by which alcohol induces these effects is unknown, decreased IGF-1 receptor production could reflect a response to lower-than-normal levels of intraovarian IGF-1. Alternatively, alcohol may interrupt the process of IGF-1 receptor gene expression by decreasing the production of IGF-1 receptor mRNA or hastening the chemical degradation of IGF-1 receptor mRNA after it is formed (Srivastava et al. 1999).

Finally, because the biological actions of IGF-1 within the ovary are modulated by IGFBPs, Srivastava and colleagues (1999) assessed ovarian protein levels of IGFBPs-3, −4, and −5, which are the most abundant forms of that protein in the ovary. Alcohol was found not to affect IGFBP-4 but to cause significantly increased levels of IGFBPs-3 and −5. IGFBPs can influence the concentration of IGF-1. For example, they can decrease the concentration of IGF-1 available for interaction with the IGF-1 receptor by binding to IGF-1 themselves. Thus, the increase of IGFBPs within the ovary may be another means by which alcohol detrimentally affects processes that lead to the attainment of sexual maturation (Srivastava et al. 1999).

In summary, alcohol can alter the IGF-1 regulatory system within the prepubertal ovary. The effects are threefold: (1) decreased IGF-1 peptide, (2) decreased IGF-1 receptor synthesis, and (3) elevated levels of specific IGFBPs. These actions may contribute to alcohol’s ability to alter prepubertal ovarian function, resulting in decreased levels of estradiol in the bloodstream at this critical time of development.

## Overview of the Ovarian Nitric Oxide System

NO is produced in cells throughout the body by three different forms of an enzyme called NO synthase (NOS). All of these forms of NOS are found in the ovary (Van Voorhis et al. 1995; Jablonka-Shariff and Olson 1997; Srivastava et al. 1997), where they initiate NO formation. Several studies have suggested that ovarian NO may play a role in the ovulatory process (Ellman et al. 1993; Ben-Shlomo et al. 1994; Shukovski and Tsafriri 1994), and that elevated NO after ovulation inhibits estradiol secretion (Van Voorhis et al. 1994, 1995; Olson et al. 1996; Srivastava et al. 1997).

## Effects of Alcohol on the Ovarian Nitric Oxide System

Because alcohol stimulates NO levels in nonovarian tissues (Davda et al. 1993; Wang and Marsden 1995; Naassila et al. 1996), Srivastava and colleagues (1999) investigated alcohol’s actions on the intraovarian NO system. Using rats, these researchers determined that 5-day exposure to alcohol caused an overall increase in NOS associated with elevated NO activity within the ovary and decreased levels of estradiol in the bloodstream. Thus, it appears that alcohol increases NO levels in the ovary as it does in other tissues. Through this mechanism, alcohol use can potentially interfere with prepubertal steroid production during a critical time of ovarian maturation when increasing levels of estradiol are important to the pubertal process (Srivastava et al. 1999).

## Conclusion

Research suggests that the normal timing and progression of puberty may be at risk in human adolescents consuming even relatively moderate amounts of alcohol on a regular basis (Dees et al. 2000). Evidence reviewed above supports a contributory role for the IGF-1 and NO systems in regulating ovarian function. Moreover, the functions of these systems can be altered by alcohol. Chronic alcohol exposure decreases production of ovarian IGF-1 and its receptor and increases ovarian production of NO. These actions suggest a combined negative effect contributing to suppressed estradiol secretion at a critical time of ovarian maturation. Thus, the effects of alcohol exposure during adolescence may result not only from disturbances of the H-P-O axis but also from altered functioning of intraovarian systems. Alcohol’s effects on intraovarian systems in mature women are unknown.

The postulated existence of multiple mechanisms whereby alcohol can delay puberty underscores the need for increased prevention and public education efforts to convince youth of the risks of drinking. Ongoing research will determine the possible long-range consequences of alcohol-induced interference with puberty.

## Figures and Tables

**Figure:**
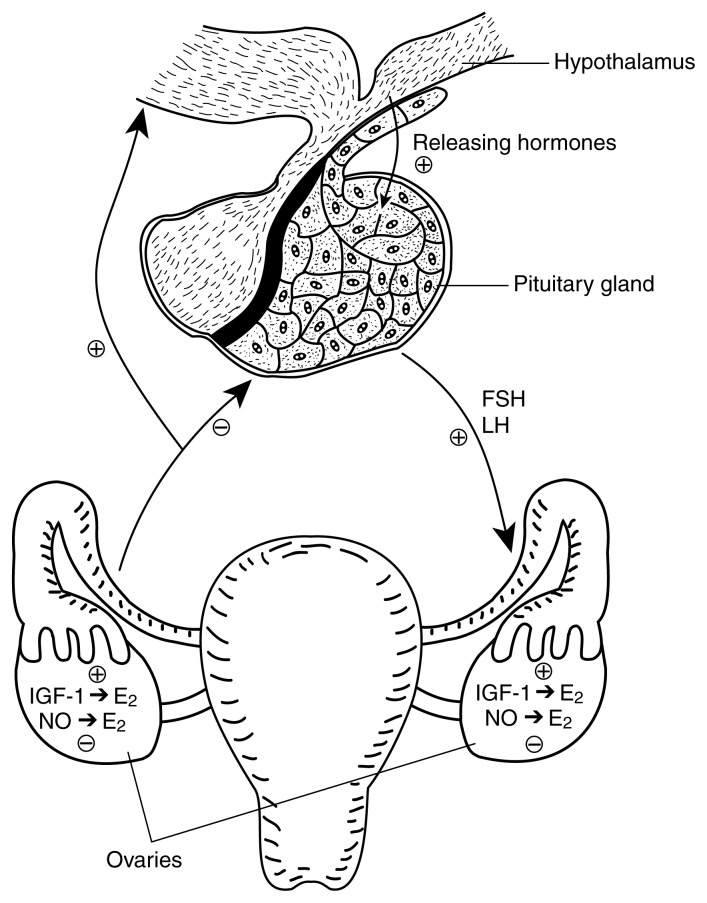
Orchestrating female puberty: The role of the H-P-O axis.^1^ The intraovarian systems described in this article supplement other reproductive control mechanisms, principally certain events governed by the activity of the H-P-O axis. As female puberty approaches, the pituitary gland begins to release increasing amounts of luteinizing hormone (LH), follicle stimulating hormone (FSH), and growth hormone (GH)^2^ in response to stimulatory “releasing” hormones produced in an area of the brain (i.e., the hypothalamus). The increased levels of these three hormones promote maturation of the ovaries. As the ovary matures, it produces and secretes increased amounts of the principle female hormone, estradiol (E_2_). Estradiol’s actions contribute to the development of both the hypothalamus and the reproductive system. Following puberty, LH and FSH continue to help regulate the events of the monthly female reproductive cycle. Ovarian production of estradiol is also stimulated by insulin-like growth factor (IGF-1) and inhibited by nitric oxide (NO). Although these substances occur elsewhere in the body as well, IGF-1 and NO produced within the ovary may play an important contributory role in ovarian growth and development. For a more detailed review of puberty-related events, see Dees and colleagues (1985). NOTE: ⊕ stimulatory effect; ⊖ inhibitory effect. ^1^The H-P-O axis consists of the hypothalamus, pituitary gland, and ovaries. ^2^GH helps regulate growth by stimulating protein synthesis, especially during adolescence.

## Footnotes

1 IGF-1 produced elsewhere in the body and released into the bloodstream also influences reproductive function (Dees et al. 1998).
